# Temporal alteration of microglia to microinfarcts in rat brain induced by the vascular occlusion with fluorescent microspheres

**DOI:** 10.3389/fncel.2022.956342

**Published:** 2022-08-03

**Authors:** Yi Shen, Jingjing Cui, Shuang Zhang, Yuqing Wang, Jia Wang, Yuxin Su, Dongsheng Xu, Yihan Liu, Yating Guo, Wanzhu Bai

**Affiliations:** ^1^Institute of Acupuncture and Moxibustion, China Academy of Chinese Medical Sciences, Beijing, China; ^2^School of Traditional Chinese Medicine, Beijing University of Chinese Medicine, Beijing, China

**Keywords:** microglia, ischemic stroke, microinfarcts, fluorescent microspheres, CX3CL1, histochemistry

## Abstract

Microglia, the resident immune cells in the central nervous system, can monitor the microenvironment and actively respond to ischemic stroke and other brain injuries. In this procedure, microglia and neurons can cross-talk *via* transmembrane chemokine, Fractalkine (CX3CL1), to impact one another. We used a rat model of multifocal microinfarcts induced by the injection of fluorescent microspheres into the right common carotid artery and examined the morphological alteration of blood vessels, microglia, astrocytes, and neurons at 6 h, 1, 7, and 14 days after modeling, along with neurobehavioral tests and the staining of CX3CL1 in this study. Our results demonstrated that in the infarcted regions, astrocytes and microglia activated in response to neuronal degeneration and upregulation of cleaved caspase-3, which occurred concurrently with vascular alteration and higher expression of CX3CL1. We provided sequential histological data to shed light on the morphological changes after modeling, which would help in the identification of new targets and the choice of the ideal time window for therapeutic intervention in ischemic stroke.

## Introduction

Stroke is a common disease of the central nervous system. Millions of people suffer from a new or recurrent stroke every year, with 87% being ischemic, caused by thromboembolic blockage of a main cerebral artery or one of its branches (Virani et al., 2021). As the second leading cause of death worldwide, stroke brings a large burden on both society and people (Ingall, 2004). To investigate the underlying mechanisms and therapeutic strategies, various animal stroke models have been developed in past decades (Sommer, 2017; Hermann et al., 2019; Kuriakose and Xiao, 2020).

An embolic stroke model could be developed by fluorescent microspheres injection, which allows it easily identify the blocked blood arteries, the distribution of the microspheres, and infarctions in histologic sections (Zhu et al., 2012; Silasi et al., 2015). Focal ischemic lesions were developed by occlusion of the unilateral common carotid artery and external carotid artery followed by the injection of fluorescent microspheres solution into the internal carotid artery. Both rats and mice were used for the surgical procedure (Himi et al., 2016; Lecordier et al., 2022; Shen et al., 2022). Different microspheres sizes and dosages led to different extent and severity of lesions (Mayzel-Oreg et al., 2004; Tsukada et al., 2018; Georgakopoulou et al., 2021).

As it was known, both the structure and function of the neurovascular unit were affected after stroke (Cai et al., 2017; Eldahshan et al., 2019). Glial cells, especially microglia and astrocytes, functioned as a component of the neurovascular unit and were essential for both brain development and damage (Wohleb, 2016; Iadecola, 2017; Tay et al., 2017; Verkhratsky and Nedergaard, 2018). Only a few minutes after ischemia events resulted in irreparable damage and subsequent neuronal death. Astrocytes and microglia activated rapidly in response to ischemic events, accompanied by vascular destruction, and then pro-inflammatory molecules and cell debris, resulting in secondary inflammatory injury (Rossi et al., 2007; Qin et al., 2019; Han et al., 2022). As phagocytes of brain, microglia cleared necrotic brain tissue and toxic cellular debris, which also contributed to inflammatory responses (Xiong et al., 2016; Amato and Arnold, 2021) and participated in the whole process of stroke.

Fractalkine (FKN, also known as CX3CL1), a chemokine that interacted with microglia and other cellular elements of the neurovascular unit, was primarily expressed on neurons (Pawelec et al., 2020). CX3CL1 regulated the activation and chemotaxis of microglia during the inflammatory process in post-ischemic brains (Liu et al., 2015; Chen et al., 2020; Ge et al., 2022). Upregulation of CX3CL1 could attract microglia to the area of inflammation, where they activated and released pro-inflammatory mediators such as cytokines, reactive oxygen species, and glutamate, leading to anti-inflammatory response and the restoration of brain function (Cardona et al., 2006; Dénes et al., 2008; Lambertsen et al., 2009; Gu et al., 2014).

In this study, we focused on the temporal alteration of microglia against the microinfarcts induced by fluorescent microspheres at sequential time points on 6 h, 1, 7, and 14 days after modeling, and tried to determine the appropriate time window for the treatment and intervention of ischemic stroke.

## Materials and methods

### Animals and surgery

Totally 37 adult male Sprague Dawley rats (6–8 weeks old, weighing 170–190 g) provided by the Institute of Laboratory Animal Sciences, Chinese Academy of Medical Sciences, China [license No. SCXK (Jing) 2019–0010], were used in this study. All animals were housed separately in a regular (12/12 h) light/dark cycle with controlled temperature and humidity and allowed free access to food and water. This study was approved by the Animal Ethics Committee of the Institute of Acupuncture and Moxibustion, China Academy of Chinese Medical Sciences (approval No. D2021-03-16-1). All the procedures were carried out in accordance with the National Institutes of Health Guide for the Care and Use of Laboratory Animals (National Academy Press, Washington, DC, USA). After 7 days of acclimatizing, animals were randomly assigned into five groups (*n* = 7 for normal, 6 h, 7, and 14 d group, and *n* = 9 for 1 d group).

In surgical approaches, respiratory anesthesia was induced by 1.5% isoflurane (Litian, Jiupai Pharmaceutical Co. Ltd., Hebei, China). The normal group received no surgical approach but anesthesia, while the other groups received surgical procedures for microspheres injection. The procedure was performed as described previously (Kisoh et al., 2017; Meloux et al., 2018; van der Wijk et al., 2020). The rats were in the supine position, the proximal sides of the right common carotid artery (CCA) and the external carotid artery were ligated with surgical silk, and the distal side of the CCA was clamped temporarily. An incision was made to insert the needle of a 1-mL syringe into the CCA. The clamp was removed before injection. A total of 600 μL of 1,000 units of microspheres with 45–53 μm in diameter (~616 μg/mL, Fluorescent Response: Peak emission of 515 nm when excited at 414 nm, UVPMSBY2-1.00, Cospheric LLC, Santa Barbara, CA, USA) suspended in 5% Dextran T-40 (Cat# D8250, Solarbio Science & Technology, Beijing, China), 1% Heparin sodium salt (Cat# 9041-08-1, Solarbio Science & Technology), and 0.02% Tween 20 (Cat# 9005-64-5, Solarbio Science & Technology) mixing with 0.1 M phosphate buffer (PB, pH 7.4) were injected during the 1 min. Then, the distal end of the common carotid artery was ligated with surgical silk and the cut was stitched. The rats were kept warm before analepsia.

### Examination of neurological functions

Zea-Longa score was used to evaluate the successful establishment of animal models and the temporal alteration of neuronal impairment, the method is as follows: 0 point, no neurological deficits, double forelimb symmetrically stretching to the ground; 1 point, contralateral forelimb weakness and torso turning to the ipsilateral side when held by the tail; 2 points, circling to affected side; 3 points, failure to bear weight on affected side; and 4 points, no spontaneous locomotor activity or barrel rolling (Longa et al., 1989; Wang L. et al., 2021; Xue et al., 2021). The evaluations were performed by three professionals who were blinded to the experimental design.

### Examination of catwalk

The Catwalk was performed as previously reported (Caballero-Garrido et al., 2017; Chen et al., 2017). Gait training was conducted in triplicate at a regular time for 5 days before modeling until the rats were able to traverse the runway without pause. The recording was performed in a quiet and clean environment without daylight; four runs with at least two complete step cycles for every rat were required. The parameter settings were as follows: The camera gain was 20, the detection threshold was 0.2, the run duration was 0.5–10 s, and the maximum speed variations was 60%. The average value of every rat was applied to data analysis.

### 2,3,5-triphenyltetrazolium chloride (TTC) staining

TTC staining was performed as previously reported (Benedek et al., 2006). Two rats in 1 d group were euthanized, the brains were dissected out quickly, then coronally sectioned into 1 mm-thick sections, dyed in 2% 2,3,5-triphenyltetrazolium chloride solution in a dark environment (Cat# G3005, Solarbio Science & Technology) at 37°C for 20 min, and fixed in 4% paraformaldehyde in 0.1 M PB for 2 h.

### Perfusions and sections

Seven rats of each group were anesthetized and euthanized by intraperitoneal injection of pentobarbital sodium (50 mg/kg, Cat# 020402, Beijing Chemical Reagent Research Institute Co., Ltd., Beijing, China) at 6 h, 1, 7, and 14 days after the operation, and transcardially perfused with saline followed with 4% paraformaldehyde in 0.1 M PB. The brains were dissected out and post-fixed in 4% paraformaldehyde in 0.1 M PB for 2 h, then cryoprotected overnight in 25% sucrose in 0.1 M PB. Every brain was cut into 80 μm coronal sections with a freezing microtome (REM-710, Yamato Koki Industrial, Osaka, Japan) and the sections were collected in order in a 12-hole Petri dish with 0.1 M PB.

### Fluorescent histochemical and immunohistochemical examinations

Fluorescent histochemical and immunohistochemical examinations were performed in the following parts, performing as described previously (Wang J. et al., 2021; Cui et al., 2022): (1) phalloidin + CD31, (2) ionized calcium binding adapter molecule 1 (Iba1) + Glial fibrillary acidic protein (GFAP) + Nissl staining, (3) CD68 + Iba1 + DAPI (4′,6-diamidino-2-phenylindole), (4) CX3CL1 + Iba1 + Nissl staining, and (5) Neuronal nuclear antigen (NeuN) + cleaved caspase- 3. Brain sections of every hole in the Petri dish was used in each examination. All sections were incubated in blocking solution containing 3% normal donkey serum (Cat# 017-000-121, Jackson ImmunoResearch, West Grove, PA, USA) and 1% Triton X-100 in 0.1 M PB for 0.5 h at room temperature. Then, they were incubated separately with primary antibodies solution at 4°C overnight: (1) goat anti-CD31 (1:500, Cat# AF3628, R&D Systems, Minneapolis, MN, USA); (2) rabbit anti-Iba1 (1:1,000, Cat# ab178847, Abcam, Cambridge, UK) + mouse anti-GFAP (1:1,000, Cat# G3893, Sigma, St. Louis, MO, USA); (3) mouse anti-CD68 (1:500, Cat# MA5-16654, Thermo Fisher) + rabbit anti-Iba1 (1:1,000, Cat# ab178847, Abcam), (4) goat anti-CX3CL1 (1:50, Cat# AF537, R&D Systems) + rabbit anti-Iba1 (1:1,000, Cat# ab178847, Abcam); and (5) mouse anti-NeuN (1:1,000, Cat# ab104224, Abcam) + rabbit anti-cleaved Caspase-3 (Asp175) (1:500, Cat# 9661, Cell Signaling).

On the next day, the samples were washed in 0.1 M PB and incubated with the secondary antibodies and biomarkers for 2 h: (1) Alexa Fluor 568 phalloidin (1:1,000, Thermo Fisher, Cat# A12380) and donkey anti-goat Alexa Fluor 488 (1:500, Cat# A11055, Thermo Fisher); (2) donkey anti-rabbit Alexa Fluor 488 (1:500, Cat# A21206, Thermo Fisher), donkey anti-mouse Alexa Fluor 594 (1:500, Cat# A21203, Thermo Fisher), and NeuroTraceTM 530/615 Blue Fluorescent Nissl Stain (1:1,000, Cat# N21482, Thermo Fisher); (3) donkey anti-rabbit Alexa Fluor 488 (1:500, Cat# A21206, Thermo Fisher), donkey anti-mouse Alexa Fluor 594 (1:500, Cat# A21203, Thermo Fisher), and DAPI (1:50,000, Cat# D3571, Thermo Fisher); (4) donkey anti-goat Alexa Fluor 488 (1:500, Cat# A11055, Thermo Fisher), donkey anti-rabbit Alexa Fluor 594 (1:500, Cat# A21207, Thermo Fisher), and NeuroTraceTM 530/615 Blue Fluorescent Nissl Stain (1:1,000, Cat# N21482, Thermo Fisher); and (5) donkey anti-rabbit Alexa Fluor 488 (1:500, Cat# A21206, Thermo Fisher), donkey anti-mouse Alexa Fluor 594 (1:500, Cat# A21203, Thermo Fisher).

After staining, all sections were washed thoroughly with 0.1 M PB and mounted on a SuperFrost Plus microscope slide (Thermo Fisher), then sealed with a coverslip in 50% glycerin before microscopic observation.

The samples were scanned with VS120 Virtual Slide System (Olympus, Tokyo, Japan), in which the representative regions were selected for further view, and recorded with a confocal imaging system (FV1200, Olympus). The fluorescent histochemical images were processed with Fiji software to obtain the percent area stained (Zanier et al., 2015; Estrada et al., 2017; Healy et al., 2018). The area ratio was calculated as the number of positive pixels to total pixels and was expressed in square micrometers. All images were processed using Adobe Photoshop CS5 (Adobe Systems, San Jose, CA, USA) and Adobe Illustration CS5 (Adobe Systems). The Imaris software (V.7.7, Bitplane, Zurich, Switzerland) was used to reconstruct three-dimensional images.

### Statistical analysis

Results were presented as the mean ± standard error of mean (SEM) with SAS version 9.4 (SAS Institute Inc., Cary, NC, USA), among which the Kruskal–Wallis test was used. Differences were considered statistically significant when *p* < 0.05.

## Results

### Neurological function score and motor ability test

**Figure 1:**
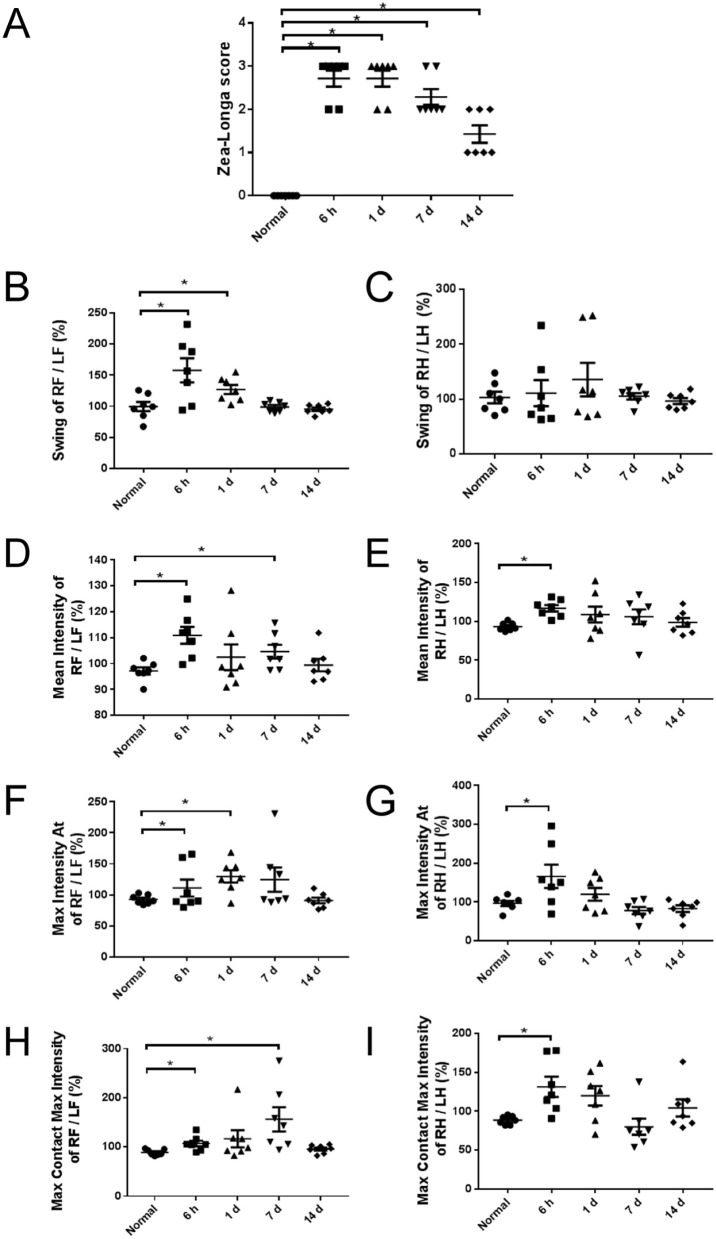
Neurological deficit of rats at different time points. **(A)** Zea-Longa score in 6 h, 1, 7, and 14 days groups increased significantly to normal group (*n* = 7). **(B–I)** The Catwalk gait analysis of groups. The pattern of gait in forelimbs and hind limbs was not completely consistent. Multi-parameters increased at 6 h after modeling and returned to the normal level at 14 days (*n* = 7). RF, right forelimb; LF, left forelimb; RH, right hind limb; LH, left hind limb. Swing (s): the duration in seconds of no contact of a paw with the glass plate. Mean intensity: the mean intensity of the complete paw. Max Intensity At (s): the time in seconds since the start of the run that the maximum intensity is measured. Max Contact Max Intensity: the maximum intensity at max contact of a paw. The intensity of a print depends on the degree of contact between a paw and the glass plate and increases with increasing weight. *p < 0.05 vs. normal group.

As early as 6 h after modeling, Zea-Longa's score raised showing that the operation was successful and the neurological function was impaired. The neurological deficit lasted for 14 days, while there was an improvement over time (Figure 1A).

The motor performance of rats is bilaterally symmetric for they are tetrapods. When a parameter was measured at both the contralateral and the ipsilateral forelimb or hind limb, a ratio of the contralateral front paw/ipsilateral front paw and the contralateral hind paw/ipsilateral hind paw were determined to detect asymmetries between right and left, which could also avoid the influence of body weights in different groups.

At 6 hours after modeling, the swing of right forelimbs/left forelimbs (Figure 1B), the mean intensity (Figures 1D,E), the max intensity (Figures 1F,G), and the max contact max intensity (Figures 1H,I) of both forelimbs and hind limbs were significantly increased. At 1 day after modeling, the swing (Figure 1B) and the max intensity (Figure 1F) of right forelimbs/left forelimbs were significantly increased, while there was no significant difference in the swing of right hind limbs / left hind limbs (Figure 1C). At 7 days after modeling, the mean intensity (Figure 1D) and the max contact max intensity (Figure 1H) of right forelimbs/left forelimbs were significantly increased. At 14 days after modeling, all parameters returned to normal levels.

### Fluorescent microsphere distribution and infarcts

**Figure 2:**
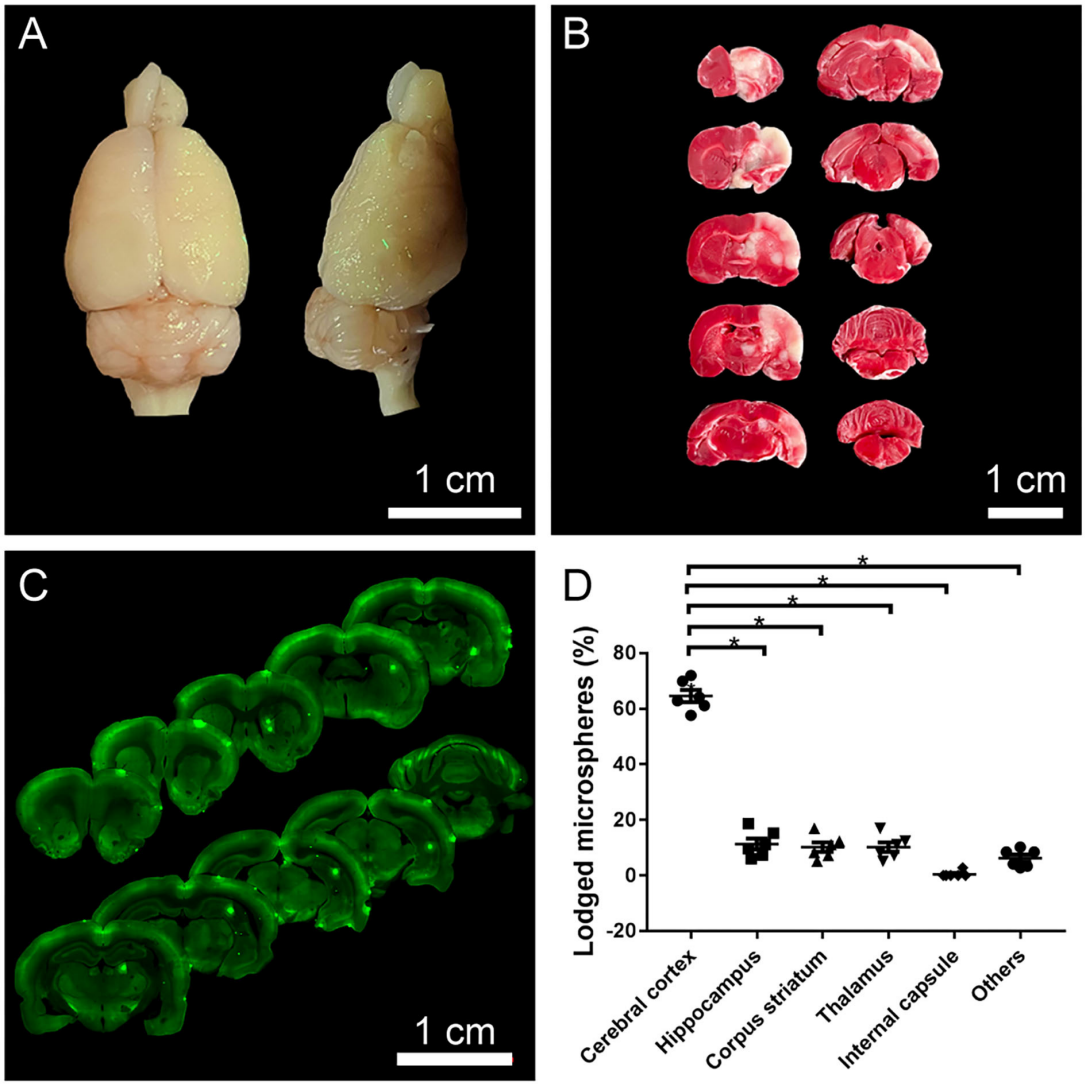
The distribution of fluorescent microspheres and infarcts. **(A)** A representative photograph from the brain surface of a rat 1 day after operation under UV illumination showing typical distribution of fluorescent microspheres (green dots) lodged in the cerebral cortex. **(B)** A representative photograph of TTC staining. The red area indicated normal areas, and the white areas indicated the infarctions, detecting that the ipsilateral cerebral cortex, hippocampus, and striatum were affected. **(C)** A representative photograph from the coronal sections of the brain with VS120 Virtual Slide System showing the lodged microspheres (green dots) in the ipsilateral cerebral cortex, hippocampus, striatum. **(D)** The distributional percentage of microspheres in the different regions and the cerebral cortex were more susceptible to damage. *p < 0.05 vs. other regions (cerebral cortex vs. hippocampus, striatum, thalamus, internal capsule, and others).

Fluorescent microspheres were directly observed on the surface of the brain under UV illumination (wavelength 395 nm), which were mainly distributed on the ipsilateral side of the operation (Figure 2A). TTC staining and coronal sections also showed a similar pattern in the distribution of microspheres and infarctions: they were mainly distributed in the ipsilateral cerebral cortex, hippocampus, striatum, and thalamus (Figures 2B,C), among which the cerebral cortex was more susceptible to be affected (Figure 2D).

### Vascular occlusion

**Figure 3:**
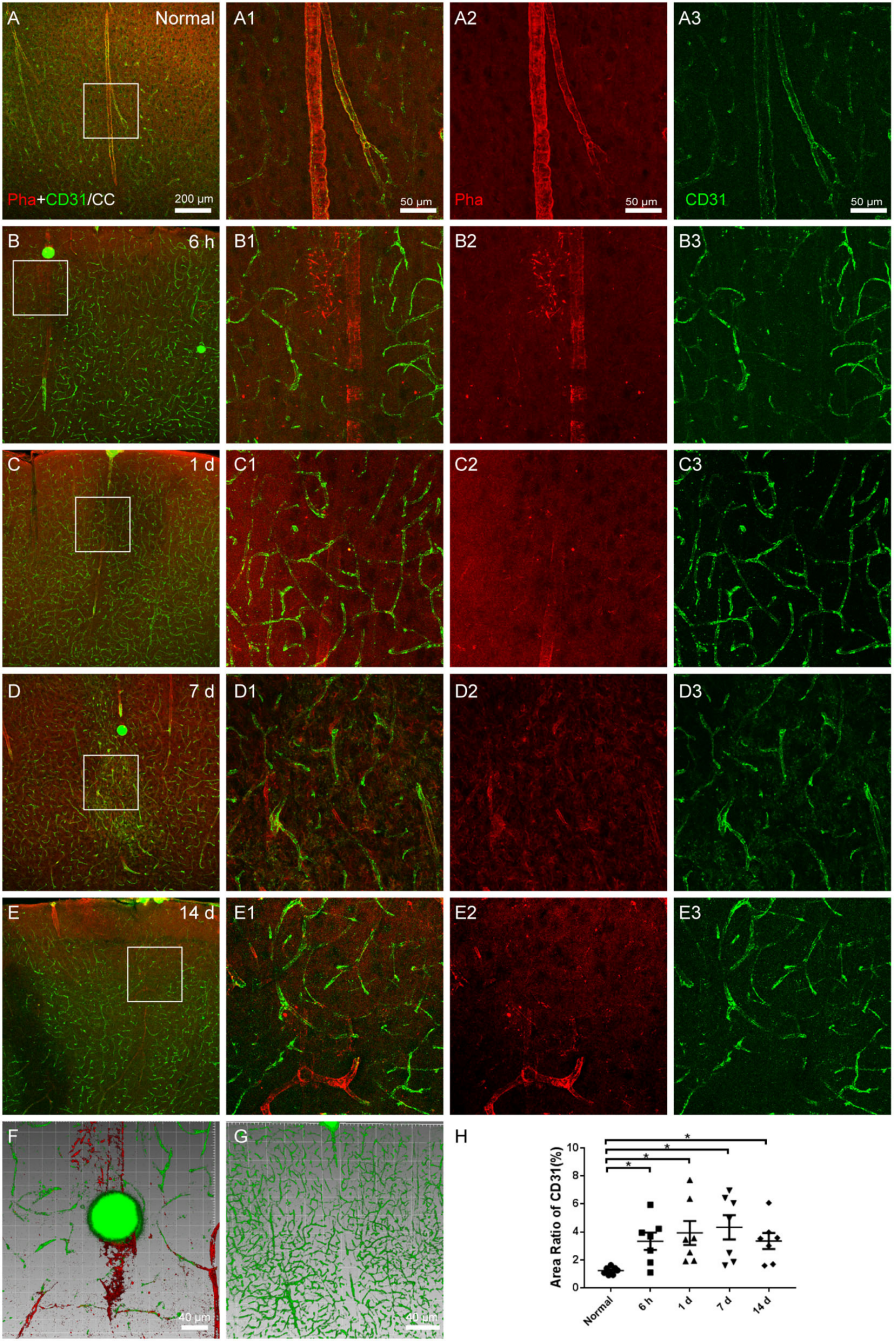
The vascular occlusion in the cerebral cortex. **(A–E)** The representative photographs from the cerebral cortex (CC) at the group of normal, 6 h, 1, 7, and 14 days, showing phalloidin (red)-labeled arteries and CD31 (green, Alexa Fluor 488)-labeled capillaries. **(A1–E1)** Magnified photographs from the box-indicated regions in **(A–E)** show the details. **(B–E)** At 6 h, 1, 7, and 14 days after the operation, the microspheres were embedded in the phalloidin-labeled arteries, following with the downstream arteries denatured or even necrotic, and CD31-labeled capillaries surrounding the infarcted vessels were significantly expressed. **(F,G)** The three-dimensional images showed that the microspheres lodged in the blood vessels, causing downstream vascular necrosis and peripheral capillary proliferation. **(H)** The area ratio of CD31 significantly increased at all time points after the operation. The vascular alteration in all model rats presented in a similar pattern (*n* = 7). The green dots in **(B–F)** were lodged in fluorescent microspheres. Scale bars: 200 μm in **(A–E)**, 50 μm in **(A1–E3)**, and 40 μm in **(F,G)**. *p < 0.05 vs. normal group.

The capillaries and arteries were marked with CD31 and phalloidin, respectively. The phalloidin-labeled arteries were continuous and undamaged under normal circumstances, with a modest expression of CD31-labeled capillaries (Figures 3A,A1–A3). Following surgery, the necrosis of blocked arteries and the proliferation of surrounding capillaries were carried out over time (Figures 3B–E). The downstream arteries became denatured or even necrotic (Figures 3B2–E2), and CD31-labeled capillaries were abundantly expressed surrounding the infarcted vessels (Figures 3B3–E3). Through the three-dimensional images, it could be seen that the microspheres lodged in the blood vessels, downstream vascular necrosis (Figure 3F, from Figure 3B), and peripheral capillary proliferation (Figure 3G, from Figure 3C).

### Astroglial and microglial activation

**Figure 4:**
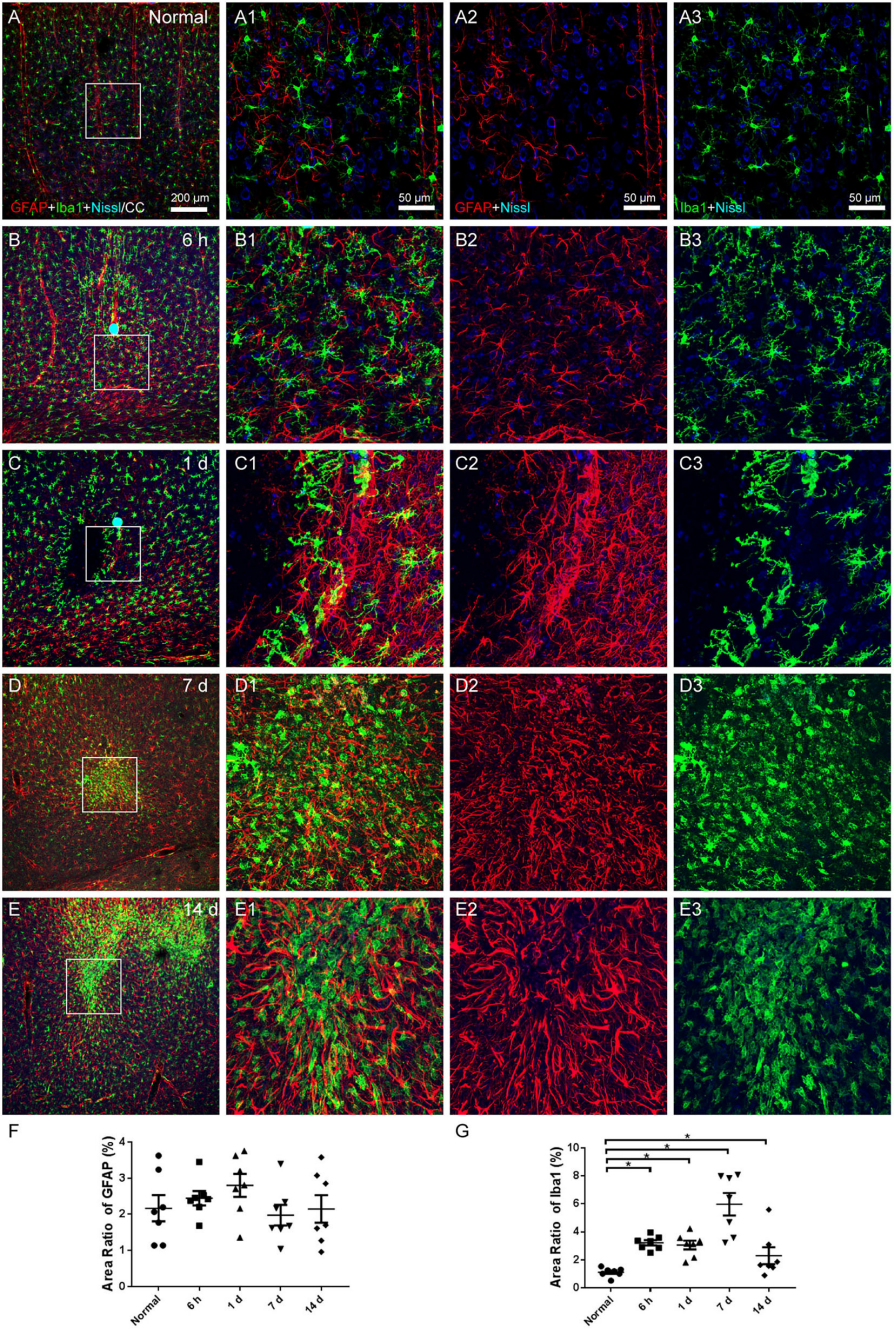
The astroglial and microglial alteration in the cerebral cortex. **(A–E)** The representative photographs from the cerebral cortex at the group of normal, 6 h, 1, 7, and 14 days, showing GFAP (red, Alexa Fluor 594)-labeled astrocytes, Iba1 (green, Alexa Fluor 488)-labeled microglia, and Nissl-labeled neurons (blue). **(A1–E1)** Magnified photographs from the box-indicated regions in **(A–E)** show the details. **(B–E)** At 6 h, 1, 7, and 14 days after the operation, astrocytes and microglia activated and gathered in the region of infarcts. **(F)** The area ratio of astrocytes showed an increasing trend after the operation, but there was no statistical significance. **(G)** The area ratio of microglia significantly increased at all time points after the operation, peaking at 7 days. The astroglial and microglial activation in all model rats presented with a similar pattern (*n* = 7). The green dots in **(B,C)** were lodged in fluorescent microspheres. Scale bars: 200 μm in **(A–E)**, 50 μm in **(A1–E3)**. *p < 0.05 vs. normal group.

Microglia were examined using Iba1 labeling, while astrocytes were evaluated with GFAP. The cerebral cortex of the normal group, which received no surgical operation, contained astrocytes surrounding the vascular walls, quiescent microglia evenly spacing throughout, and healthy Nissl-labeled neurons (Figures 4A,A1–A3). At 6 h, 1, 7, and 14 days after the operation, astrocytes and microglia activated and gathered in the region of infarcts. The processes of astrocytes were thicker, the microglia were with enlarged cell bodies and shorter processes, and the expression of Nissl-labeled neurons decreased (Figures 4B–E). The area ratio of astrocytes showed an increasing trend after the operation, but there was no statistical significance (Figure 4F). The area ratio of microglia considerably increased at all time points after the operation (Figure 4G).

### The activated state of microglia

Iba1 has been the most widely used marker for the immunohistochemical analysis of microglia, which could stain ramified, activated, amoeboid, and dystrophic microglia. It cannot be utilized to differentiate between various functional microglial morphologies, while ramified microglia exhibit a high level of CD68 expression, which is a sign of phagocytic activity.

**Figure 5:**
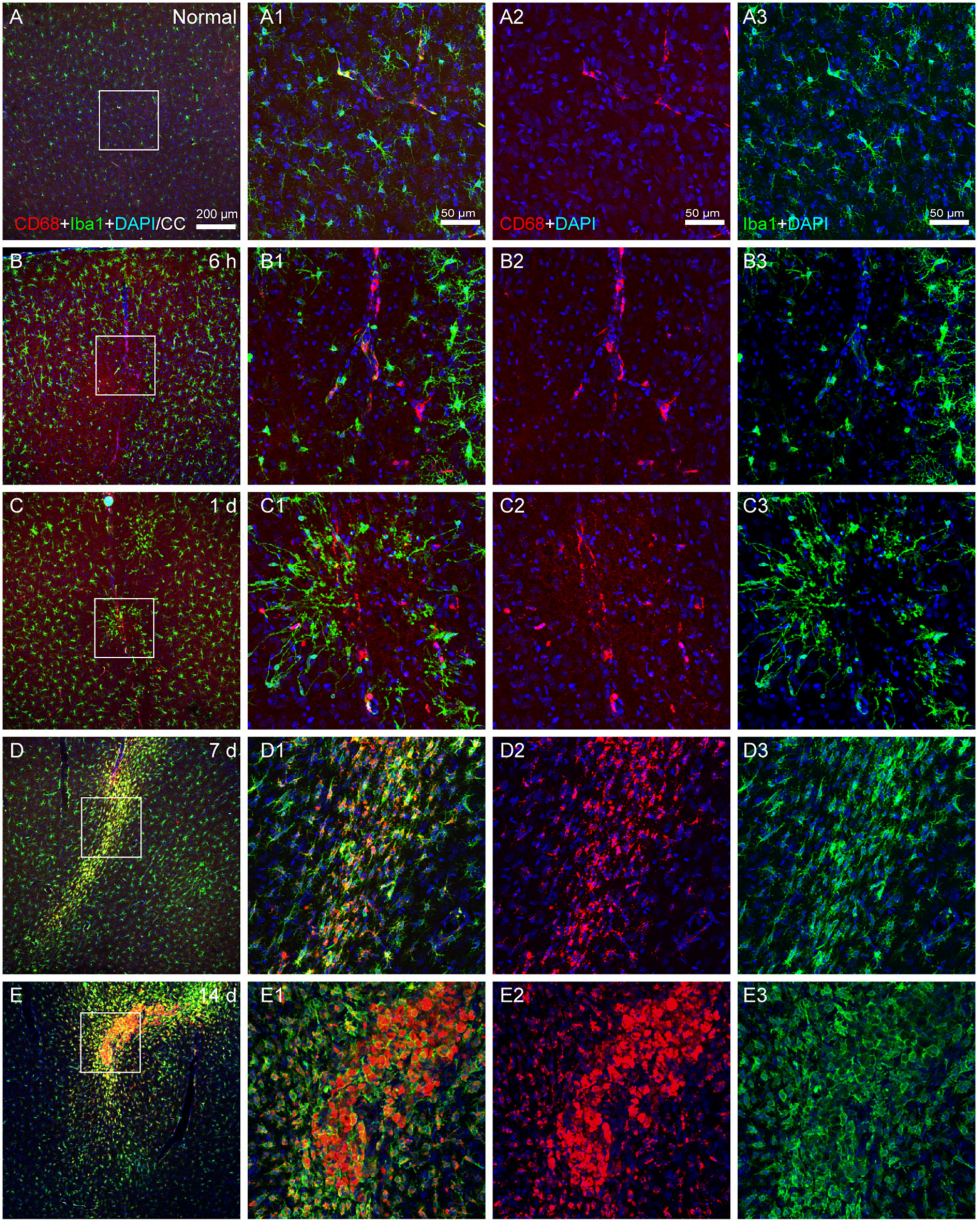
The activated state of microglia. **(A–E)** The representative photographs from the cerebral cortex at the group of normal, 6 h, 1, 7, and 14 days, showing CD68 (red, Alexa Fluor 594)-labeled and Iba1 (green, Alexa Fluor 488)-labeled cells. **(A1–E1)** Magnified photographs from the box-indicated regions in **(A–E)** show the details. **(B–E)** Following surgery, microglial phenotypes with phagocytic activity could be identified at 6 h, 1, 7, and 14 days. The alteration in all model rats presented in a similar pattern (*n* = 7). The green dot in **(C)** was lodged in fluorescent microspheres. Scale bars: 200 μm in **(A–E)**, 50 μm in **(A1–E3)**.

The number of Iba1- and CD68-colabeled microglia was low in the normal state (Figures 5A,A1–A3), but increased following the operation. There were a few CD68 (+) Iba1 (–) cells on the vascular walls at 6 h and 1 day after modeling, together with a sparse population of Iba1- and CD68-colabeled microglia (Figures 5B,C). At 7 and 14 days after modeling, a large number of Iba1- and CD68-colabeled microglia accumulated in the infarcted area (Figures 5D,E).

### Microglial and CX3CL1 alteration

**Figure 6:**
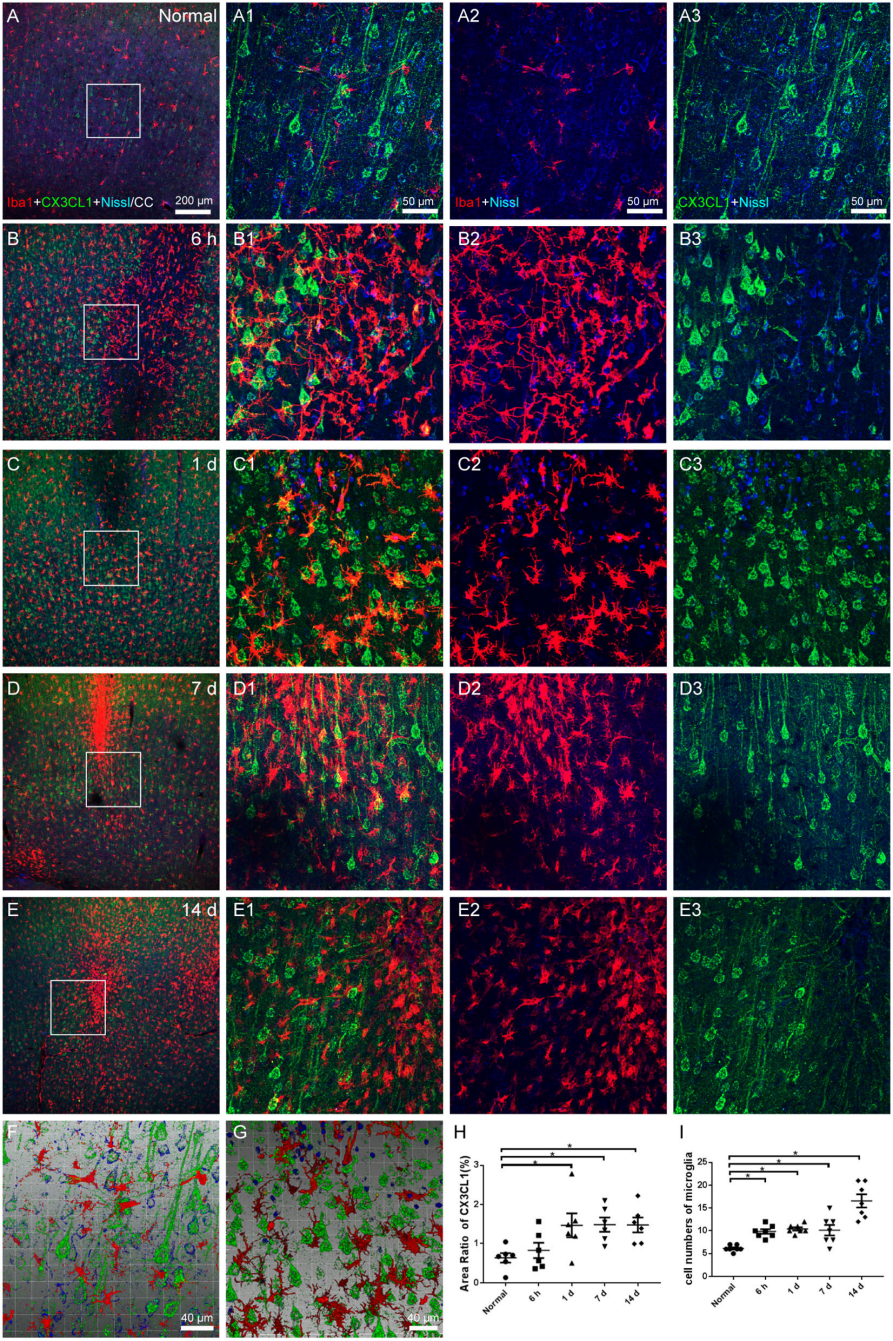
Microglial and CX3CL1 alteration in cerebral cortex. **(A–E)** The representative photographs from the cerebral cortex in groups of normal, 6 h, 1, 7, and 14 days, showing the Iba1 (red, Alexa Fluor 594)-labeled microglia and CX3CL1 (green, Alexa Fluor 488)-labeling. **(A1–E1)** Magnified photographs from the box-indicated regions in **(A–E)** show the details. **(A–E)** Quiescent microglia were evenly distributed with the processes interacting with CX3CL1 expressed on Nissl-labeled neurons in the normal group. Modeling was followed by the activation and accumulation of microglia around the increased neurons that expressed CX3CL1. **(F,G)** The three-dimensional images showed the increased contact of microglia and neurons after modeling. **(H)** The area ratio of CX3CL1 significantly increased at 1, 7, and 14 days after the operation. **(I)** Quantitation of microglia surrounding CX3CL1-labeled neurons with a radius of 100 μm in ischemic penumbra. The alteration in all model rats presented in a similar pattern (*n* = 7). Scale bars: 200 μm in **(A–E)**, 50 μm in **(A1–E3)**, 40 μm in **(F,G)**. *p < 0.05 vs. normal group.

Iba1-labeled microglia were uniformly dispersed and in a resting state under normal circumstances, interacting with CX3CL1 expressed on a portion of Nissl-labeled neurons (Figures 6A,A1–A3). The pattern of active microglia in the infarcted area resembled earlier findings, CX3CL1 expression increased (Figures 6B–E,H), and the contact between microglia and neurons increased (Figure 6F, from Figure 6A1; Figure 6G, from Figure 6C1; Figure 6I). Additionally, it should be noted that the neurons expressing CX3CL1 in the penumbra appeared to be more than those in the core of the infarction, which may be due to neuronal survival and compensatory collateral circulation after modeling.

### Neuronal degeneration and distribution

**Figure 7:**
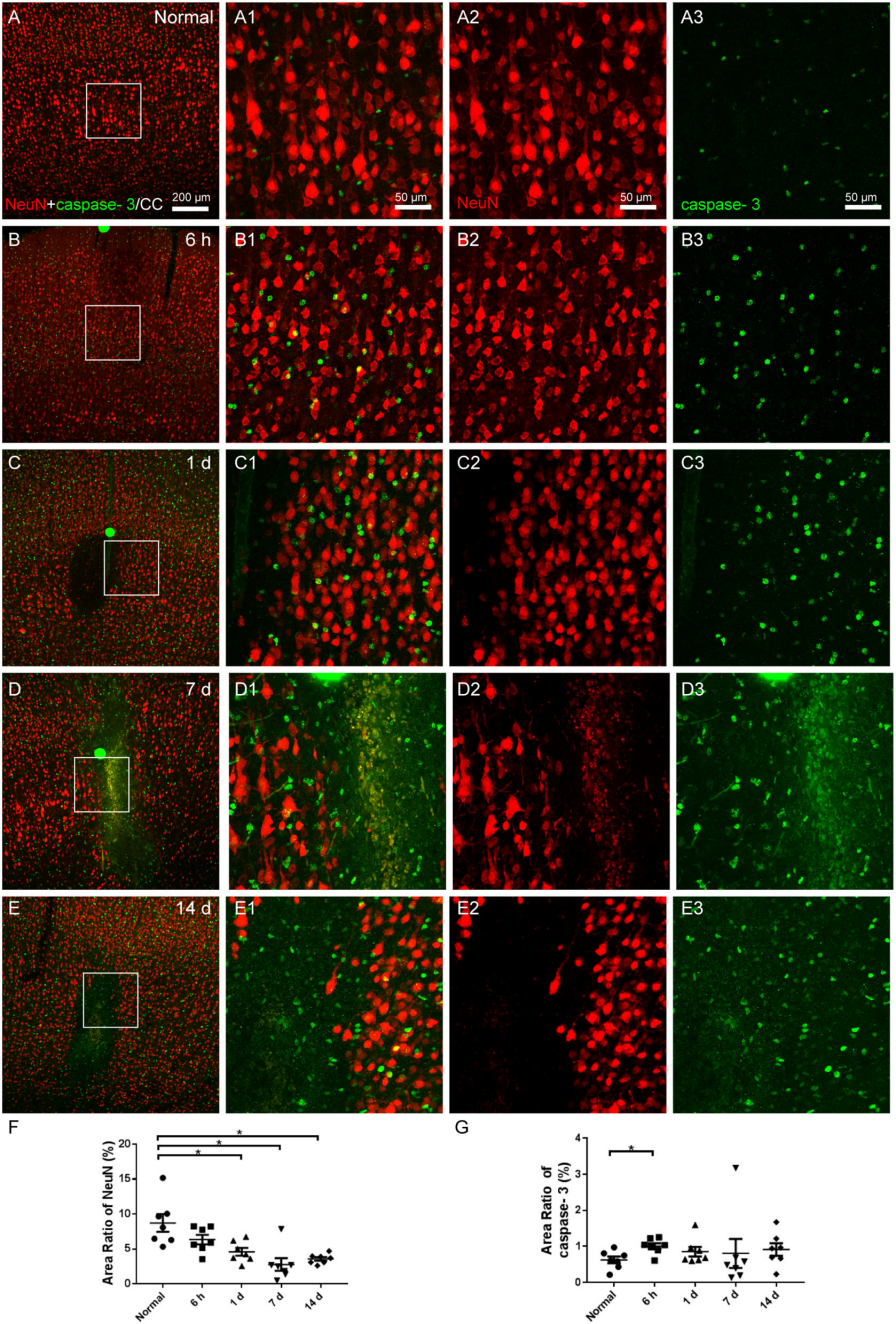
The neuronal degeneration in the cerebral cortex. **(A–E)** The representative photographs from the cerebral cortex in groups of normal, 6 h, 1, 7, and 14 days, showing the NeuN (red, Alexa Fluor 594)-labeled neurons and cleaved caspase-3 (green, Alexa Fluor 488) labeling. **(A1–E1)** Magnified photographs from the box-indicated regions in **(A–E)** show the details. **(A–E)** After the operation, neuronal shrinkage and loss showed up in the area of the infarcts, and the amount of cleaved caspase-3 increased. **(F)** The area ratio of neurons showed an increasing trend of neuronal apoptosis after the operation. **(G)** The area ratio of caspase-3 significantly increased at 6 h after the operation. The neuronal degeneration in all model rats presented with a similar pattern (*n* = 7). The green dots in **(B–D)** were lodged in fluorescent microspheres. Scale bars: 200 μm in **(A–E)**, 50 μm in **(A1–E3)**. *p < 0.05 vs. normal group.

Morphological changes of neurons were assessed with NeuN and apoptosis process with cleaved caspase-3 staining. The neurons in the cerebral cortex of the normal group were equally distributed, and the expression of cleaved caspase-3 was low (Figures 7A,A1–A3). Although we captured images of a few colabeled cells, neuronal atrophy and cell loss emerged in the area of the infarctions at 6 h, 1, 7, and 14 days following the operation, and the expression of caspase-3 increased (Figures 7B–E). The area ratio of neurons showed an increasing trend of neuronal loss (Figure 7F), while the area ratio of caspase-3 significantly increased only at 6 h after the operation (Figure 7G).

## Discussion

In this study, we described the neurobehavioral changes and pathological properties of multiple microinfarcts at different time points after modeling produced by the injection of fluorescent microspheres into the unilateral common carotid artery. The alteration of neurons, microglia, and astrocytes in a time-dependent pattern may be potentially helpful to determine the optimal time window for the treatment of ischemic stroke.

### The rodent model of microspheres injection

Ischemic stroke in humans is a highly complicated and heterogeneous disorder, whose severity depends on the etiology and localization of the infarct, duration, and blood pressure, as well as age, sex, and genetic background (Sommer, 2017). Although there is still a disconnect between experimental models and human experience, most of our understanding of ischemic stroke is established in the experimental stroke models (Dirnagl and Endres, 2014). For the stroke models, mice and rats are typically used to mimic the stroke of human being, and each model has its advantages and disadvantages, such as middle cerebral artery occlusion (MCAo) (Longa et al., 1989; Boutin et al., 1999; Shah et al., 2019) and embolic occlusion (Watson et al., 1985; Niessen et al., 2003; Bacigaluppi et al., 2010; Silasi et al., 2015; Zhang et al., 2015). In general, these models limited the blood supply to specific regions of the brain. Considering the advantages and disadvantages of each model, the model of multiple microinfarcts induced by the vascular occlusion with fluorescent microspheres was selected to systematically analyze in this study.

Due to the visibility of fluorescent microspheres in tissue sections, it is convenient to observe the distribution of infarct regions. Similar to our previous study on the mice (Shen et al., 2022), the fluorescent microspheres were mainly distributed on the perforating arteries nearby cerebral cortex, hippocampus, striatum, and thalamus ipsilateral to the side of injection, and caused the ischemic lesions (Bralet et al., 1983, 1987; Tsukada et al., 2018). The degree of damage was directly correlated with the diameter of microspheres and their number in the injected solution (Rapp et al., 2000, 2003). Although this model has been frequently applied in pathological observation and therapeutic intervention (Zhang et al., 2019; Georgakopoulou et al., 2021), in this study, we observed the temporal alteration of blood vessels, astrocytes, microglia, and neurons in the infarct regions, especially on the cerebral cortex at different time points after modeling from the perspective of histochemistry.

### Technological and design limitations

Currently, studies have demonstrated the repeatability and animal survival of the model, making it appropriate for preparing the experimental model of focal ischemia. This study still has its limits, though. We concentrated on temporal morphological changes, but more comprehensive research using various qualitative and quantitative techniques would be needed to address the topic of the neurovascular unit, including the temporal alteration of different functional microglial phenotypes (Lier et al., 2021), how other components changed at various time points, such as endothelial cells, vascular smooth muscle cells, and pericytes, and how they interacted with one another. Whether CX3CL1 could mediate microglia polarization to influence the outcome of stroke is also a problem. Additionally, gender and age should be focused in further research. Researchers have continuously focused on how gender and age affect ischemic stroke: Clinical and rodent experiments identified that young women had a lower incidence and better prognosis of stroke, while this trend reversed after menopause (Branyan and Sohrabji, 2020; Tang et al., 2022). The comprehensive research of stroke on gender, age, and other related diseases may be a potential orientation in the future. For instance, a study found that multifocal microinfarcts aggravated cognitive decline more potently in young male mice of Alzheimer's disease compared to young females (Lecordier et al., 2022).

### Neurobehavioral alteration at different time points after ischemia stroke

Zea-Longa score and Catwalk gait analysis were used in this study to access the behavioral alteration. Zea-Longa score, as one of the most commonly used neurological functional methods in experimental stroke models, could effectively evaluate neurological deficit. The time parameters (Swing and Max intensity at) and pressure parameters (Mean intensity and max contact max intensity) of Catwalk could reflect the changes in gait symmetry. We found that there was some inconsistency between the trend of the Zea-Longa score and the gait parameters. At 6 h and 1 day after modeling, the gait parameters changed significantly, showing the asymmetric limb dysfunction in the acute phase of cerebral ischemia, which was consistent with the Zea-Longa score. However, at 14 days following surgery, there were no significant differences in gait parameters between modeling groups and normal groups, even if there were still differences in Zea-Longa scores. Researchers have suggested that more severe cerebral ischemia lesions, such as the MCAo model, may result in obvious limb asymmetry, whereas photothrombosis and microsphere-injection models may not (Bärmann et al., 2021). The neural networks in the spinal cord could also play a role in regulating rhythmic movements, such as gait, flight, swimming, or breathing (MacKay-Lyons, 2002; Hamers et al., 2006). Thus, other methodologies should be chosen or the applicability of various behavioral tests to models should be completely explored in future studies.

### Gial and neuronal alteration at different time points after ischemia stroke

The neurobehavioral failure resulted from the neuronal degeneration of the brain following ischemic events, which could be found in the TTC staining and fluorescent histochemical images.

TTC is one of the most common histochemical stains used to assess the cerebral injury. In ischemic tissue, lack of TTC staining is considered “infarcted” and defined as core while viable tissue is stained red. Normal zone, ischemic penumbra, and ischemic core could be observed in the affected cerebral hemisphere *via* TTC staining, which was related to the blood supply of the internal carotid artery, and matched up with the distribution of microspheres in the brain slices observed under the fluorescence microscope.

As a marker of apoptosis activation, cleaved caspase-3 increased significantly as early as 6 h after the operation. We observed few colabeling of cleaved caspase-3 and neurons, which may result from the irreversible neuronal damage in the ischemic core within minutes of ischemia and hypoxia. That is why time is of the essence in ischemic intervention. Other markers and methods for detecting apoptosis should also be applied in the future. Except for executing cell apoptosis, caspase-3 also promotes the activation of microglia (Burguillos et al., 2011; Radak et al., 2017).

Microglia and astrocytes are in a resting condition in the healthy cerebral cortex (Lynch et al., 2010; Domercq et al., 2013; Zanier et al., 2015). In the early stage (6 h) after modeling, astrocytes and microglia were activated, which could be characterized by larger cell bodies and thicker processes and gathering in the infarcted regions. It was also consistent with our previous results in the mice model (Shen et al., 2022). The activation lasted 14 days after modeling in the infarcted regions with microspheres. Various damage-related molecular patterns and cytokines activated astrocytes after ischemia events, which caused considerable alterations in their morphological and functional features (Li et al., 2022). Besides, they could also regulate the balance of synaptic glutamate and respond in time to the changes of ions and metabolism of neurons. Afterward, a glial scar developed, impeding the functional recovery in the chronic phase (Li et al., 2022; Takahashi, 2022).

Microglia, the tissue-resident macrophages of the central nervous system, play significant immune roles to support the development, maintenance, homeostasis, and repair of the brain (Nimmerjahn et al., 2005), also modulate the development and functions of neurons and glial cells through both direct and indirect interactions (Werneburg et al., 2017; Greenhalgh et al., 2020; Mehl et al., 2022). We examined functionally activated microglia by CD68, which was indicative of phagocytic activity. Microglia that were CD68-labeled activated at the late stage of 7 and 14 days, as opposed to those that were Iba1-labeled early on. This may be explained that CD68 was also expressed in macrophages, neutrophils, and monocytes after the blood-brain barrier destroyed.

Chemokines have a variety of roles in the brain, facilitating cell-cell communication and regulating processes including neuroprotection after injury (Araujo and Cotman, 1993), of which CX3CL1 is a crucial member. CX3CL1 is considered to be expressed mainly on neurons, and its receptor CX3CR1 is expressed on microglia (Tran and Miller, 2003). The way they communicate is that the neuronal cell bodies-expressed CX3CL1 is contacted by microglial processes (Harrison et al., 1998; Hatori et al., 2002; Cserép et al., 2020).

During ischemic brain injury, CX3CL1 upregulates on neuronal cells, leading to chemotaxis of microglia, in which the microglial processes increasingly contact with the neurons. We counted the microglia numbers surrounding the CX3CL1-labeled neurons to evaluate the contact, to demonstrate the cross-talk between them, with reference to a previous study (Meng et al., 2019). We observed that CX3CL1 expression of neurons increased in the infarcted regions of the cerebral cortex, microglia activated and gathered, which contacted with the CX3CL1-labeled neurons more. The enhanced expression should restrict inflammation in favor of the functional repair of brain tissue and have a net anti-inflammatory effect (Sheridan and Murphy, 2013). Besides, we found there were several CX3CL1-positive cells without Nissl staining in modeling groups. Previous studies pointed out that astrocytes could also express CX3CL1 at lower levels than neurons, particularly in response to pro-inflammatory signals, which occurred in disorders such as stroke, multiple sclerosis, and Alzheimer's disease (Sheridan and Murphy, 2013).

## Summary

**Figure 8:**
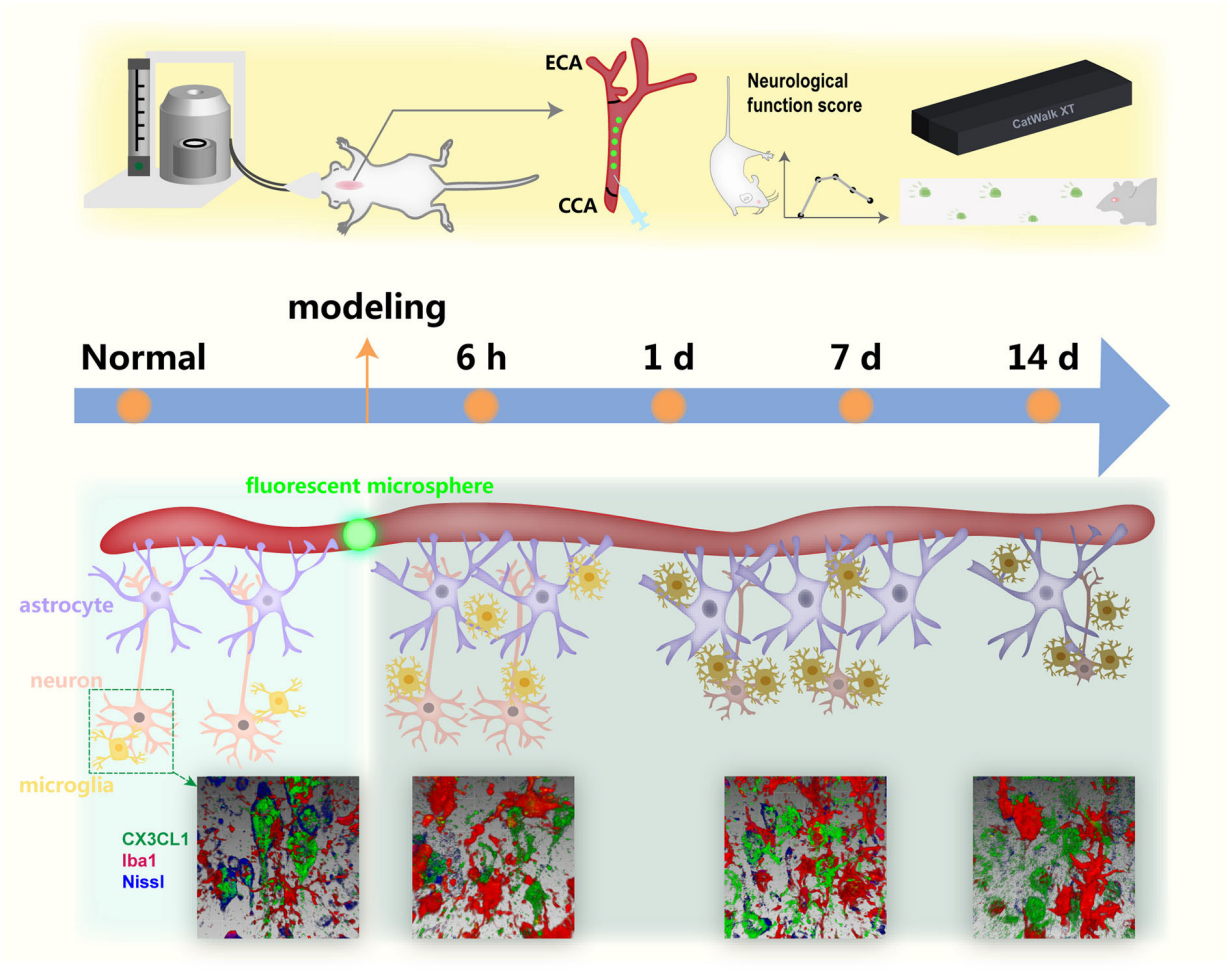
A simplified illustration of the temporal alteration in the infarcted regions with lodged fluorescent microsphere in the cerebral cortex.

The multicellular changes in the microsphere-injection model at different time points have been not completely clear by now. We focused on the morphological alteration of microglia, astrocytes, and neurons at 6 h, 1, 7, and 14 days after modeling, combined with neurobehavioral tests and the staining of CX3CL1, providing the evidence for selecting the therapeutic time window and potential target by revealing the temporal changes (Figure 8).

## Data availability statement

The raw data supporting the conclusions of this article will be made available by the authors, without undue reservation.

## Ethics statement

The animal study was reviewed and approved by Animal Ethics Committee of the Institute of Acupuncture and Moxibustion, China Academy of Chinese Medical Sciences.

## Author contributions

YSh and WB: study conception and design. YSh and SZ: model preparation. YSh, JC, JW, and YG: behavior tests and histochemical staining. YW, YSu, and DX: data analysis and figure preparation. YSh, YL, and WB: manuscript drafting. All authors approved the final manuscript.

## Funding

This study was supported by the CACMS Innovation Fund, No. CI2021A03407 (to WB), the Project of National Key R&D Program of China, No. 2019YFC1709103 (to WB), the National Natural Science Foundation of China, Nos. 81774432 (to JC), 81774211 (to WB), 82004492 (to JW), and 81801561 (to DX), and the Fundamental Research Funds for the Central Public Welfare Research Institutes of China, Nos. ZZ13-YQ-068 (to JC), ZZ14-YQ-032 (to JW), and ZZ14-YQ-034 (to DX).

## Conflict of interest

The authors declare that the research was conducted in the absence of any commercial or financial relationships that could be construed as a potential conflict of interest.

## Publisher's note

All claims expressed in this article are solely those of the authors and do not necessarily represent those of their affiliated organizations, or those of the publisher, the editors and the reviewers. Any product that may be evaluated in this article, or claim that may be made by its manufacturer, is not guaranteed or endorsed by the publisher.
